# Supplementary material for: Patterns of Intron Gain and Loss in Fungi
Source: PLoS Biol. 2004 Nov 30;2(12):e422. doi: 10.1371/journal.pbio.0020422 (PMC532390; doi:10.1371/journal.pbio.0020422)
Supplement: Table S1 — Also available at http://genes.mit.edu/NielsenEtAl/. (4.3 MB ZIP). [file pbio.0020422.st001.zip › NielsenEtAl/html/243.html]

AN3708.1.NCU06876.1.MG00872.1.FG07949.1


```
 CLUSTAL W (1.82) Multiple Sequence Alignments - Introns Inserted


Sequence 1: AN3708.1	188 aa
Sequence 2: FG07949.1	128 aa
Sequence 3: NCU06876.1	136 aa
Sequence 4: MG00872.1	141 aa
Alignment Length: 196 aa
Number Identitical Residues: 69 aa
Alignment Score (without introns) 3295


MG00872.1 	MQKEGGRIRAEEKEL0TIRTG~------------~DIPSFKLFENEKVFAFLDINPLSRG
NCU06876.1	MASSATCIFCK----~-IVKG1------------~EIPCFKLVETEKTLAFLDIQPLSRG
FG07949.1 	---MSNCIFCK----~-IIKG1------------~DIPSFKLFESDKTLAFLDIGPLSKG
AN3708.1  	---MAACIFCR----~-IIKG~KQHSLLALEEAR1EIPSFKLFESDKVFAFLDIQPLSRG
          	    .  * ..      * .* .. :  : ..:  :**.***.*.:*.:***** ***:*

MG00872.1 	HS0LVIPKFHGEKLHDIPDDSLNEIL0---------------------------------
NCU06876.1	HA0LVIPKHHGEKLADIPDDSLSELL~---------------------------------
FG07949.1 	HA0LVIPKYHGAKLADIPDEDLAEVL0---------------------------------
AN3708.1  	HA0LVIPKYHGAKLTDIPDDHLTEIL0YLAGYTPPPPGPSPPNALPVSIHIHGFSNGPSY
          	*: *****.** ** ****: * *:*   :. :......:...: . :    . :...: 

MG00872.1 	----------PVAKKIAQAVGAKDYNILQNNGAIAHQVVPH0VHFHM0IPKPNETEGLGV
NCU06876.1	----------PIAKKLAIASGAVDYNILQNNGKLAHQEVGH~VHVHM~IPKPNEKEGLIV
FG07949.1 	----------PVLKKIVNATGATDYNILQNNGRIAHQEVDH0-----~IPKPNNEEGLGV
AN3708.1  	WRELTDDDKQPVAKKIAQVTGATDFNVLQNNGRIAHQVVDH0VHFHM0IPKPNEEEGLGI
          	  . :.....*: **:. . ** *:*:***** :*** * *       *****: *** :

MG00872.1 	KWPMQETDMDKLKALFEDIKSKM
NCU06876.1	GWPQQATDMEGLKKLHAEIKEKL
FG07949.1 	TWPTKPANMEQLKAYCEEIKSKI
AN3708.1  	GWPAQATDMDKLKEYYESIKAKM
          	 ** : ::*: **    .** *:
```
